# Effects of preoperative anxiety levels and the D-type personality on propolol injection pain

**DOI:** 10.14744/nci.2020.61214

**Published:** 2022-02-10

**Authors:** Okkes Hakan Miniksar, Ahmet Yuksek

**Affiliations:** Department of Anesthesiology and Reanimation, Yozgat Bozok University, Yozgat, Turkey

**Keywords:** Anxiety, injection, pain, personality, propofol, venous cannulation pain

## Abstract

**Objective:**

Propofol injection pain (PIP) is a common condition in anesthesia practice and can be detrimental for patients. In this study, we aimed to investigate the effects of preoperative anxiety, depression levels, and D-type personality trait on PIP and to determine predictive factors.

**Methods:**

Sixty-eight patients who underwent elective septorhinoplasty under general anesthesia were analyzed. The effects of various factors (preoperative anxiety, depression, D-type personality, venous cannulation pain, gender, and age) on the incidence of PIP were assessed. To identify risk factors associated with PIP, multivariate logistic regression analysis was performed.

**Results:**

The incidence of PIP was significantly higher in patients who had preoperative anxiety and venous cannulation pain and who are Type D personality and female. Preoperative anxiety (β, 2.914; p=<0.001), Type D personality (β, 2.225; p=0.022) and venous cannulation pain (β, 1.590; p=0.043) were identified as independent risk factors for development of PIP. Depression, general anesthesia history, marital status, smoking, education status, and age were not significant as risk factors for the PIP.

**Conclusion:**

In addition to the physical factors that can predict PIP in anesthesia application, we believe that the presence of preoperative anxiety, Type D personality, and venous cannulation pain is significant, and it will be useful to apply preventive treatments for injection pain.

**P**ropofol is the most commonly used intravenous (IV) anesthetic agent for anesthesia induction and maintenance during surgeries [1]. However, the pain associated with propofol injection in adults is a distressing condition that affects between 28% and 90% of all patients, is known to reduce patient satisfaction and safety [1, 2]. According to patients, propofol injection pain (PIP) was stated to be the seventh most important problem in clinical anesthesia experience [2].

Studies have stated that there are many factors responsible for PIP, but the specific mechanism has not been revealed yet. It has been claimed that propofol-related PIP has two basic mechanisms. First, the phenol groups contained in the propofol directly irritate the skin, mucous membranes, and venous wall intimacy, and stimulate the nociceptors and free nerve endings, and the second; activates the quinine-calicrine system by indirectly affecting the endothelium of the propofol [3–5]. Factors affecting the incidence of pain in propofol injection; injection site, injection rate, vessel size, propofol concentration in the aqueous phase, buffering effect of blood, sex and use of various drugs together [6]. Therefore it was recommended to evaluate clinical factors affecting PIP in each patient prior to induction [7, 8].

Psychogenic problems such as anxiety, depression, somatoform disorders, and fear of pain are frequently observed in patients undergoing elective surgery. Among these, preoperative anxiety and pain sensitivity has been the most interesting factors [9]. Preoperative anxiety activates the release of neuroendocrine mediators related to stress response and negatively affects anesthetic approaches, surgical recovery, and postoperative pain levels [10].

Pain sensation is specific to the individual’s personal experience is due to psychological factors [11, 12]. The personality, which is the sum of inborn and societally developed characteristics, has a very important place in the perception of pain [11]. The Type D personality, or the “distressed or anxious personality” is characterized by a combination of negative affectivity and social inhibition [9, 12]. Individuals with type D personality are more depressed and angry than other people and these people have been shown to feel pain more often and more severely [13]. For these reasons, it is important to examine psychogenic factors such as anxiety, depression, and type D personality that might affect PIP. This can help develop PIP prevention strategies for high-risk patients.

There is no published data on the effects of preoperative anxiety levels and D type personality trait on PIP. The primary aim of our study is to examine the effects of preoperative anxiety, depression levels and D-type personality trait on PIP. Our secondary goal is to identify predictive factors for PIP.

## Materials And Methods

### Study population

This prospective observational study was carried out between February 1 and April 15, 2020 in the Medical Faculty Hospital of Yozgat Bozok University. Ethics committee approval of this study was given by the Clinical Research Ethics Committee of Yozgat Bozok University (Reference number: KAEK-189_2020.01.22_04).

Sixty-eight patients, aged 18 to 55, American Society of Anesthesiologists Physical Status (ASA) I–II, who gave written informed consents and underwent elective septorhinoplasty surgery with propofol induction were included in the study. Exclusion criteria were poor understanding or communication difficulty, mental illness, hypersensitivity to study drugs, history of chronic pain or those taking regular analgesics preoperatively, those who had applied more than once IV cannulation, those with a known psychiatric illness and drug use.

**Highlight key points**

• Propofol injection pain (PIP) is a common condition in anesthesia practice.
• Preoperatif anxiety, Type D personality and venous cannulation pain were identified as independent risk factors for development of PIP.
• Evaluation of this factors that can predict PIP is important for the application of preventive treatments for PIP.

### Procedure

When the patients were referred to the anesthesia outpatient clinic, type-D personality scale was used to assess the personality trait, and the Hospital Anxiety and Depression Scale (HADS) was used to determine the level of anxiety and depression 1 hour before the operation. During the preoperative visit, patients signed the informed consent, and were informed about the visual analogue score (VAS) and verbal rating scale (VRS) for pain scoring. We used VRS since there would be loss of consciousness after propofol administration. None of the patients received premedication. When patients were brought to the operating room, nurses who did not participate in the study placed the 20-gauge IV cannula in a vein in the dorsum of the hand and the normal saline infusion was started. Patients were asked to estimate the maximum intensity of pain associated with this procedure on a horizontal VAS scale (0–10). Patients were then divided into two according to VAS levels of <3 or ≥3. Those with VAS ≥3 were considered as the group with venous cannulation pain [14]. All study medications were kept at room temperature and used within 30 minutes. Twenty-five percent of the calculated 2 mg.kg^-1^ propofol dose (Propofol Lipuro 1%®, Braun, Germany) was given IV during 10 seconds after 3 min preoxygenation. The patients were asked to evaluate their injection site pain by using the VRS (0=none, 1=mild, 2=moderate, and 3=severe). In our study, pain was classified as presence of significant pain (VRS score ≥2) and absence of pain (VRS score <2) according to severity. The remaining dose of propofol was administered after initial evaluation. Prior to tracheal intubation, 1 μg/kg of fentanyl and 0.6 mg/kg rocuronium was administered to all patients. Anesthesia was continued with sevoflurane in 50% oxygen/nitrogen oxide. 

Hospital Anxiety and Depression Scale (HADS) was developed by Zigmond and Snaith (1983) [15] and its Turkish version was found valid and reliable by Aydemir et al. (1997) [16]. The scale consists of 14 items that are divided into two sub-classes: anxiety (7 items) and depression (7 items). Each item is scored between 0 and 3. The cutoff scores of the HADS subscales are 10 for anxiety and 7 for depression. Having a higher score than these cutoff values indicates that the person is in the risk group for the relevant subscale.

Type-D personality scale (DS14) was developed by Denollet (2005) [17] for use in epidemiological and clinical studies. It consists of two subscales: negative affectivity (NA) and social inhibition (SI), each consisting of 7 items. Each item is scored between 0 and 4, and participants are considered to have D type personality traits if the total score for both subscales is 10 or above. Alçelik et al. [18] demonstrated the validity and reliability of the Turkish version of DS14.

### Statistical Analyses

IBM SPSS for Windows, version 23.0 package program (SPSS Inc., Chicago, Ill, USA) was used for statistical analysis. Descriptive values were given as mean and frequency. Relationships between the incidence of PIP and risk factors such as anxiety, depression and D-type personality traits, gender, general anesthesia history, marital status, smoking, educational status, age, and venous cannulation pain, was examined by Chi-Square test or Fisher’s exact test. The variables found important in univariate analyzes were analyzed with the multivariate binary logistic regression (BLR) backward Wald model, and variables found to be statistically significant were shown in the table. In the regression analysis, the fit of the model was examined by Omnibus Tests (p<0.05), goodness of fit by Hosmer and Lemeshow tests (p>0.05), and impact coefficient by Nagelkerke R square. Results for p<0.05 were considered statistically significant.

The main point we examined was the relationship between PIP and preoperative anxiety level. Sample size estimation was based on the results of the study on pain sensitivity (r2=0.469, P=0.000) by Kil et al. [19] We calculated that a total of 53 patients were needed for such an r value (0.4) with bidirectional, 0.05 error level, and 85% power. However, 67 patients were included in our study, which could allow up to 20% withdrawal from the study. Sample size estimation was performed by using G*Power (version 3.1.9.6; Kiel, Germany) software.

## Results

A total of 68 patients (male/female: 27/41) with a mean age of 36.98±1.26 years were included in this study (Table 1). 

**Table 1.** Groups’ age, gender and ASA values

Age (years)	36.98±10.32
Gender (male/female)	27/41
ASA class (1/2)	33/35

All data were presented as mean±SD or number of the patients. SD: Standard deviation; ASA: American Society of Anesthesiologists.

In the comparative univariate analysis of risk factors for PIP, preoperative anxiety, Type D personality, female gender and venous cannulation pain were statistically significant differences (Table 2). These factors were included in the logistic regression analysis, which indicated that preoperative anxiety, Type D personality and venous cannulation pain were independent risk factors (Table 3). These important variables explain 64.9% (Nagelkerke R Square=0.649) of the occurrence of PIP. These independent risk factors are preoperative anxiety (β, 2.914; SE, 0.745; 95% CI, 0.013–0.234; p=<0.001), D type personality trait (β, 2.225; SE, 0.972; 95% CI, 0.016–0.726; p=0.022) and venous cannulation pain (β, 1.590; SE, 0.787; 95% CI, 1.048–22.198; p=0.043). Besides, the rate of reporting pain among women was found to be significantly different from men (χ^2^, 13.229; p=<0.001) in the univariate analysis (Table 2), still gender was not an independent risk factor in the BLR analysis (Table 3).

**Table 2.** Univariate analysis of risk factors for propofol injection pain

Factors	Pain (+)	Pain (-)		Total		
		%	%	%	χ^2^	p*
Preoperative anxiety				
Present	89.20	25.80	60.30	28.305	<**0.001**
Absent	10.80	74.20	39.70		
Type D personality				
Present	94.60	61.30	79.40	11.443	**0.001**
Absent	5.40	38.70	20.60		
Venous cannulation pain				
Present	94.60	48.40	73.50	18.504	<**0.001**
Absent	5.40	51.60	26.50		
Gender				
Female	59.50	16.10	39.70	13.229	<**0.001**
Male	40.50	83.90	60.30		
Depression				
Present	18.90	35.50	26.50	2.328	0.123
Absent	81.10	64.50	73.50		
History of general anesthesia				
Present	54.10	58.10	55.90	0.110	0.740
Absent	45.90	41.90	44.10		
Marital status				
Married	64.90	67.70	66.20	0.062	0.803
Single	35.10	32.30	33.80		
Smoking				
Present	40.50	25.80	33.80	1.636	0.201
Absent	59.50	74.20	66.20		
Educational status				
College graduate	64.90	58.10	61.80	0.330	0.565
Not a college graduate	35.10	41.90	38.20		
Age				
40>	56.80	67.70	61.80	0.862	0.353
40<	43.20	32.30	38.20

*: p-value for Chi-square test.

**Table 3.** Analysis of factors affecting propofol injection pain using binary logistic regression model

	β	SE	Sig.	Exp (B)	95% CI for EXP (B)	
					Lower	Upper
Preoperative anxiety	2.914	0.745	0.000	0.540	0.013	0.234
Type D personality	2.225	0.972	0.022	0.108	0.016	0.726
Venous cannulation pain	1.590	0.787	0.043	3.970	1.048	22.198
Gender	1.439	0.782	0.091	4.217	0.630	28.246
Constant	5.900	2.659	0.026	365.152		

Hosmer and Lemeshow Test=0.793; Nagelkerke R Square=0.649; β: Standardized regression coefficient; SE: Standard error; B: Nonstandardized regression coefficient; CI: Confidence interval.

## Discussion

The aim of this study was to determine the relationship between preoperative anxiety, depression, and D-type personality trait with propofol injection pain and to determine conditions that are risk factors for PIP. According to our results, we found preoperative anxiety, D-type personality, and venous cannulation pain as independent risk factors for PIP.

Propofol is widely used in clinical practice, and pain due to injection is one of the most common side effects of this IV agent [1]. Various strategies such as venous occlusion with a tourniquet, alternative propofol formulations, and pretreatment with pharmacological agents have been applied to reduce PIP. However, none of them has completely eliminated PIP and it has been reported that preventive applications should be multimodal [7, 8, 20].

In general, age and gender are believed to be predictors for postoperative pain and analgesic consumption. In the incidence of PIP, while some studies did not report gender differences [21] most studies found that younger patients and female patients were more susceptible to pain [7]. In a meta-analysis study examining clinical predictors of acute pain, factors such as young age, female gender, anxiety, and depressive symptom history were found to be predictors or pain [22]. It is stated that the reason for gender differences detected in PIP might be because men have larger vessels than women and their pain sensitivity is different [7, 23]. In our study, the incidence of pain was higher in female patients, and this was consistent with previously published studies. Erkılıç et al. [24] reported that the most important risk factors affecting preoperative anxiety in surgical patients in Turkish society were young age, female gender, and low education level. We thought that high education level would help during the patients’ preoperative preparations and reduce preoperative anxiety. However, in our study, we did not find any significant relationship between education level and PIP. Similarly, Wells et al. [25] reported that the level of education was not a predictor of preoperative anxiety. Although gender is unchangeable risk factor, we still think that can be used to predict PIP and personalize preventive analgesia requirements in the preoperative period. Another factor, smoking, was not found to be related to PIP in our study. However, according to different studies, it has been stated that smoking is a negative factor for postoperative pain control [26].

The fear of venous cannulation is one of the important anxiety situations in patients undergoing surgery and may cause severe pain in most patients. However, vascular puncture for venous cannulation is a necessary procedure for safe and effective anesthesia [14, 27]. Studies have reported that venous cannulation pain is a predictor of postoperative acute pain [14] and correlates with preoperative anxiety [27]. In these studies, it was found that venous cannulation pain was associated with anxiety and female gender, and patients with VAS> 2 experienced more postoperative pain [14, 27]. Consistent with the literature, it was found that venous cannulation pain in the preoperative period was a predictor of PIP.

Although there are various studies that investigated the causes of PIP, studies investigating psychogenic factors are limited. Vaughn et al. [28] reported that psychogenic factors were stronger predictors of pain perception than physiological factors because psychogenic factors are thought to affect patients’ pain threshold and postoperative pain sensitivity [29]. To the best of our knowledge, there are no other studies that showed a significant relationship between preoperative anxiety level and severity of PIP. Dedic et al. [30] reported that when administered orally the sedative agent midazolam, which they applied preemptively, significantly reduced injection pain and recall compared NSAIDs. In the same study, no difference was found between the anxiety levels of the patients. Other studies have also reported that premedication with midazolam reduced PIP [31, 32]. However, in these studies, the preoperative anxiety levels of the patients were not determined and therefore, anxiety was not evaluated as a risk factor for PIP. Another study reported that high-dose ketamine pretreatment reduced PIP due to central sedative/analgesic effects [33]. The effects of preoperative anxiety on intraoperative anesthetic consumption have also been documented. This might be due to hemodynamic and metabolic effects resulting from neuroendocrine response secondary to anxiety [10, 34].

An older study reported that there was no reduction in the incidence of PIP due to premedication [35]. However as the majority of studies suggest PIP can be reduced by decreasing the anxiety of the patients with appropriate sedative premedication. The fact that people with high preoperative anxiety level feel PIP more acutely shows that it may be an important treatment target in patients with injection pain. Good communication with the patient, reassuring the patient, and the application of premedication with anxiolytics if not contraindicated are approaches that can relieve preoperative anxiety and in turn reduce postoperative pain [29]. Randomized studies are needed to evaluate the effect of reducing preoperative anxiety as a replaceable risk factor on PIP and other surgical pain.

Type D personality has been reported as a psychosocial factor associated with negative clinical outcomes [36–38]. Studies have shown that type D personality is associated with certain psychiatric symptoms such as depression and anxiety, and that these patients feel postoperative pain more severely [18, 22, 36, 37]. In a different study, it was shown that D-type personality traits were not associated with musculoskeletal pain, but rather with depression [38]. Another study reported that even mild depressive symptoms result in poor pain control [22]. Therefore in our study, we hypothesized that patients with type D personality would also feel PIP more severely. In this study, D-type personality was found to be a risk factor for PIP as well as preoperative anxiety. Compared to the literature, we believe that the relationship between acute pain and D-type personality should be the subject of more studies.

When the relations of psychogenic factors with PIP were examined in our study, we found that preoperative anxiety was a strong predictor of PIP. These results supported the theory that the only predictor of postoperative pain is situational anxiety and that increased anxiety reduces the patient’s pain threshold [28]. More studies on more patients are needed to investigate the effects of changing psychogenic factors on PIP. We also think that our study will provide crucial data for future pain control studies.

In conclusion, pain perception is individual and is associated with psychogenic risk factors in addition to tissue damage. Our results showed that D-type personalities, patients with high venous cannulation pain and high preoperative anxiety levels were more susceptible to pain after IV propofol injection. We also found that depression was not associated with PIP. Thus, we believe that in clinical anesthesia practice, evaluation of preoperative anxiety and venous cannulation pain in addition to physical factors that can predict PIP is important for the application of preventive treatments for propofol injection pain.
